# Evaluation and failure analysis of four commercial deep learning‐based autosegmentation software for abdominal organs at risk

**DOI:** 10.1002/acm2.70010

**Published:** 2025-02-13

**Authors:** Mingdong Fan, Tonghe Wang, Yang Lei, Pretesh R. Patel, Sean Dresser, Beth Bradshaw Ghavidel, Richard L. J. Qiu, Jun Zhou, Kirk Luca, Oluwatosin Kayode, Jeffrey D. Bradley, Xiaofeng Yang, Justin Roper

**Affiliations:** ^1^ Department of Radiation Oncology and Winship Cancer Institute Emory University Atlanta Georgia USA; ^2^ Department of Medical Physics Memorial Sloan Kettering Cancer Center New York New York USA; ^3^ Department of Radiation Oncology Icahn School of Medicine at Mount Sinai New York New York USA; ^4^ Department of Radiation Oncology University of Pennsylvania Philadelphia Pennsylvania USA

**Keywords:** artificial intelligence, autosegmentation, organs at risk, outlier analysis

## Abstract

**Purpose:**

Deep learning‐based segmentation of organs‐at‐risk (OAR) is emerging to become mainstream in clinical practice because of the superior performance over atlas and model‐based autocontouring methods. While several commercial deep learning‐based autosegmentation solutions are now available, the implementation of these tools is still at such a primitive stage that acceptance criteria are underdeveloped due to a lack of knowledge about the systems’ segmentation tendencies and failure modes. As the starting point of the iterative process of clinical implementation, this study focuses on the outlier analysis of four commercial autocontouring tools for the abdominal OARs.

**Materials and methods:**

The autosegmentation software, developed by Limbus AI, MIM Contour ProtégéAI, Radformation AutoContour, and Siemens syngo.via, were used to segment 111 patient cases. Geometric segmentation accuracy was quantitatively compared with clinical contours using the dice similarity coefficient (DSC) and 95% Hausdorff distance (HD95). The outliers from quantitative evaluations of each software were analyzed for the liver, stomach, and kidneys with the possible causes of outliers summarized into six categories: (1) difference in contouring style or guideline, (2) image acquisition and quality, (3) abnormal anatomy of the OAR, (4) abnormal anatomy of abutting organs/tissues, (5) external/internal devices, and (6) other causes.

**Results:**

For the liver segmentation, the most prominent cause of discrepancies for Limbus, which occurred in four of its six outliers, was the existence of biliary stent or internal/external biliary drain as well as the resulting pneumobilia. Siemens included the abutting organs that shared CT numbers similar to those of the liver in 5/8 outliers. 12 of 13 Radformation's liver segmentation outliers included the heart and/or stomach while MIM not only included the stomach in the presence of barium in 5/11 outliers, but also produced fragmented contours in 5/11 other cases. Only Limbus and Radformation provided stomach segmentation, and imaging with barium contrast directly caused incomplete stomach delineation in 10/12 Limbus outliers and 21/25 Radformation outliers. As for the kidneys, Radformation and Siemens consistently followed the RTOG contouring guidelines, whereas the institutional contours excluded the renal pelvis in some cases, resulting in 19/25 Radformation outliers and 18/23 Siemens outliers. By contrast, Limbus contours appeared to follow different contouring guidelines that exclude the renal pelvis. Fragmented kidney contours were found in 10/15 Limbus outliers and 25/26 MIM outliers. The ones in MIM were directly linked to the use of IV contrast in imaging, but there was not enough evidence to identify the origin of Limbus's fragmented contours.

**Conclusion:**

The causes of the segmentation outliers of the four commercial deep learning‐based autocontouring solutions were summarized for each OAR. This work can help the vendors improve their autosegmentation software and also inform the users of potential modes of failure when using the tools.

## 1 INTRODUCTION

Delineation of target volumes and organs‐at‐risk (OAR) is an important step in radiotherapy treatment planning. The manual delineation is a very time‐consuming task^1^, ^2^ and subjective by nature. Despite consensus contouring guidelines,^3^, ^4^, ^5^ there are intra‐observer and inter‐observer variations,^1^, ^6^, ^7^, ^8^ causing inconsistencies in the delineation, which can ultimately affect the quality of the patient's treatment plan.

In order to minimize human workload and improve contouring consistency, great efforts have been made in autosegmentation (or autocontouring). Deep learning‐based autosegmentation techniques are emerging to become mainstream due to their significant improvements over traditional approaches,^1^ and multiple commercial software options are now available.

However, implementation of these commercial software tools is challenging. Before a deep learning‐based autosegmentation software can be routinely utilized clinically, extensive evaluation should be performed during the software commissioning with case‐specific quality assurance (QA) required to identify and then correct any segmentation errors.^9^, ^10^ The implementation of such tools is still at the initial stage, where there is a lack of acceptance criteria due to insufficient performance tests, and the performance of deep learning‐based autosegmentation is constantly updated with the inclusion of a wider variety of training data. Currently, most vendors only provide a pre‐trained segmentation tool, and there is a lack of transparency among these commercial products regarding the deep learning architectures, training dataset, performance evaluation, and possible limitations.^11^ Therefore, as the starting point of this iterative process of implementation, the performance of the commercial software should be extensively studied, and a list of segmentation outliers should be collected during performance evaluation. The outlier analysis can reveal instances where auto‐contours significantly deviate from the normal performance of the algorithm. Deep learning‐based autocontouring is becoming more mainstream in clinical practice, therefore, it is critical for the end users to be aware of failure modes. Furthermore, results from this study can inform the vendors on model weaknesses that may be addressed by future model training.

As institutions started to implement commercial autosegmentation solutions in the clinic, investigations on their performance have been reported. There have been studies that performed an extensive evaluation of Limbus Contour and Siemens autosegmentation for various disease sites^12^, ^13^, ^14^, ^15^ and studies where Limbus, MIM, or Radformation were implemented and compared with other commercial solutions.^16^, ^17^, ^18^ For abdominal OARs specifically, there have been quantitative and qualitative evaluations of MIM Contour Protégé, RayStation, and Limbus Contour, and MVision AI,^19^, ^20^ but no failure analyses were conducted. Heilemann et al. included a clinical guideline on autosegmentations from Limbus, RayStation, and Annotate based on the interdisciplinary discussion. However, the notes were very brief and could not cover the variety of segmentation failures that they encountered.^16^

Therefore, an in‐depth retrospective study was conducted to uncover the most prominent root causes of the segmentation outliers generated by four commercial deep learning‐based autosegmentation software, Limbus Contour, MIM Contour ProtégéAI, Radformation AutoContour, and Siemens syngo.via with 111 patients for the OARs in the abdomen disease site. Our work is focused on the limitations of the autosegmentation software. To the best knowledge of the authors, this work is the first to have an outlier analysis of any commercial deep‐learning autocontouring software for the abdominal OARs as well as the first outlier analysis of MIM and Radformation software for any disease sites.

## 2 MATERIALS AND METHODS

### 2.1 Clinical contours and autocontours

This study was approved by the Institutional Review Board. The OARs studied in this work include the liver, stomach, and kidneys from a total of 111 patients. The studied commercial software includes Limbus Contour (v1.6, MIM Contour ProtégéAI (v7.1.4, Radformation AutoContour (v1.7.11), and Siemens syngo.via (V50B). The number of auto‐segmentations produced by different software ($N_{auto}$) was detailed in Table 1 for each OAR. $N_{auto}$ was different for each software and each OAR due to incomplete data collection. Geometric evaluations and subsequent outlier analysis were performed for a software tool only if the autosegmentation of the OAR was supported by the software.

**TABLE 1:** Geometric evaluation of deep learning autosegmentation software for abdominal OARs (liver, stomach, and kidneys). The median (μ) and interquartile width ($q_{w}$) of DSC and HD95 (unit: Mm) were calculated for each OAR, and the number of total autosegmentation cases ($N_{\mathrm{auto}}$) and the number of outliers ($N_{\mathrm{outlier}}$) were provided for each software tool.

Disease Site	OAR	Evaluation	Limbus	MIM	Radformation	Siemens
Abdomen (111 cases)	Liver	$N_{auto}$	68	105	105	99
		${\left[\mu ,q_{w}\right]}_{DSC}$	[0.950, 0.029]	[0.938, 0.036]	[0.919, 0.054]	[0.952, 0.018]
		${\left[\mu ,q_{w}\right]}_{HD95}$	[5.86, 4.07]	[7.50, 7.12]	[11.34, 11.68]	[4.98, 2.10]
		$N_{outlier}$	6	11	13	8
	Stomach	$N_{auto}$	63		101	
		${\left[\mu ,q_{w}\right]}_{DSC}$	[0.871, 0.115]		[0.829, 0.174]	
		${\left[\mu ,q_{w}\right]}_{HD95}$	[12.65, 20.80]		[17.80, 34.09]	
		$N_{outlier}$	12		25	
	Kidneys (left and right)	$N_{auto}$	130	208	210	204
		${\left[\mu ,q_{w}\right]}_{DSC}$	[0.940, 0.031]	[0.891, 0.118]	[0.944, 0.030]	[0.935, 0.028]
		${\left[\mu ,q_{w}\right]}_{HD95}$	[3.86, 3.70]	[7.25, 8.07]	[3.34, 4.01]	[4.01, 3.55]
		$N_{outlier}$	15	26	25	23

### 2.2 Geometric evaluations

Two geometric metrics were used to assess the similarity between the clinical contours and the autocontours were dice similarity coefficient (DSC) and 95th percentile Hausdorff distance (HD95). While volume‐based metric DSC is a good measure of the overall segmentation accuracy, it is not sensitive to small discrepancies from the reference. On the other hand, the distance‐based metric HD95 can capture the boundary deviations that might be overlooked by volume‐based metrics, and therefore is a good complement to DSC.^11^ As a paired organ, the left and right kidneys were evaluated separately since function may be uneven and therefore laterality errors could be important, but the evaluation results were combined.

### 2.3 Outlier analysis

The outliers are determined using quantitative evaluations. The DSC and HD95 were calculated for each OAR and each autosegmentation tool. The 25th ($q_{25}$) and 75th ($q_{75}$) percentiles of DSC and HD95 values were computed. Cases with DSC values below $q_{25}-w*q_{w}$ or HD95 values above $q_{75}+w*q_{w}$ were considered outliers, where w is the multiplier length and $q_{w}$ is the interquartile width ($q_{w}=q_{75}\;-q_{25}$). The w was set at 1.5 for the liver and kidneys and 0.5 for the stomach in an effort to increase the number of organ specific outliers for the subsequent analysis. The outliers were reviewed by a medical physics resident, a medical physicist, and a radiation oncologist, and the possible causes of the outliers were summarized into six categories: (i) difference in contouring styles or guidelines, (ii) image quality and acquisition (e.g., CT with and without contrast), (iii) abnormal anatomy of the OAR, (iv) abnormal anatomy of nearby organs/tissues, (v) the presence of external and internal devices, and (vi) other causes. Outliers can be attributed to one or multiple causes.

## 3 RESULTS

### 3.1 Geometric evaluation

**FIGURE 1:**
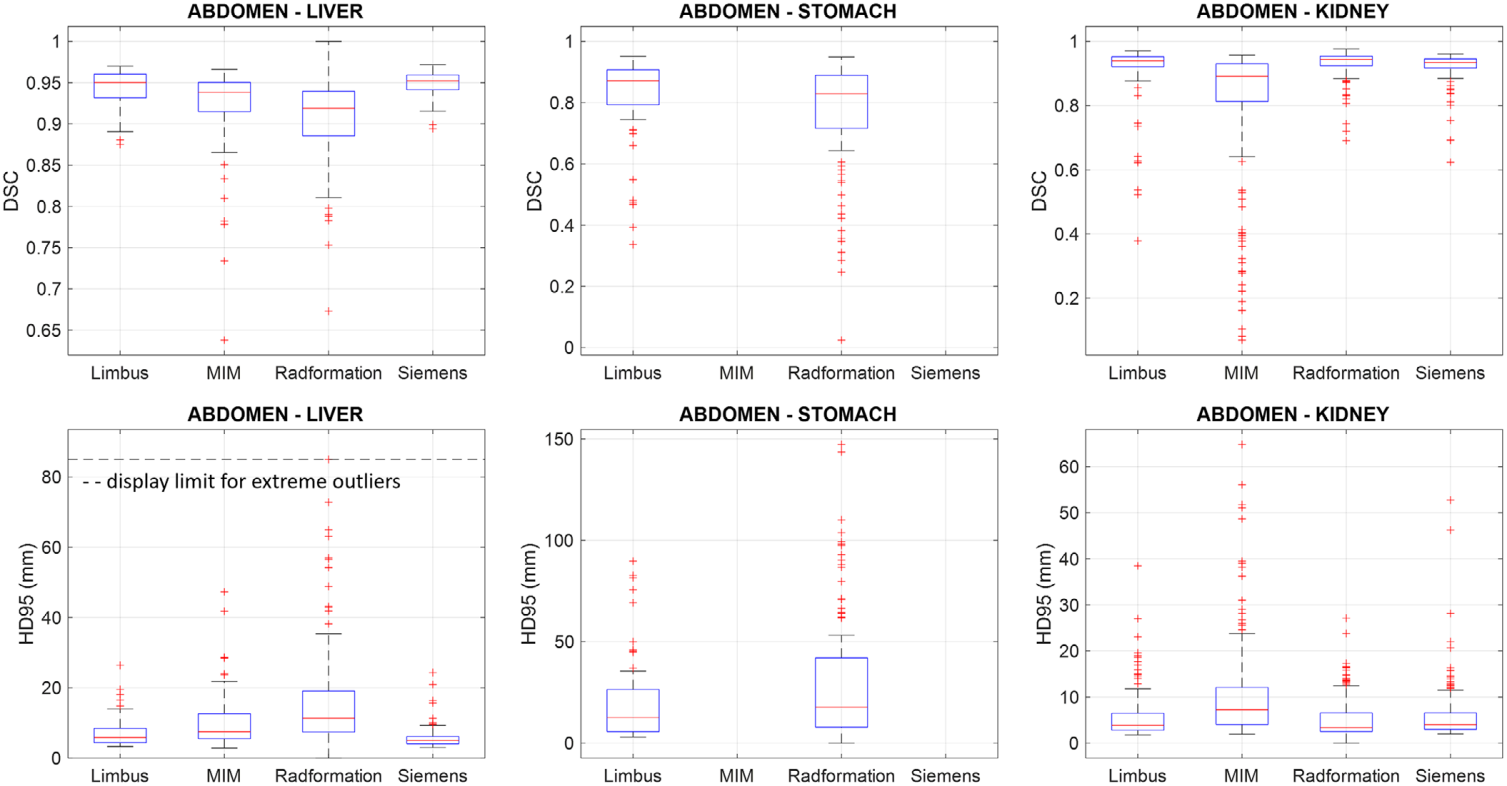
DSC and HD95 evaluations of deep learning autosegmentations for the liver, stomach, and kidneys. The HD95 display limit for extreme outliers in liver was set at 85 mm.

The geometric evaluations of deep learning autosegmentation software for the abdominal OARs are displayed in boxplots in Figure 1. In order to provide better visualization in the case of extreme outliers, a display limit was inserted in the HD95 plot for the liver. The median (μ) and interquartile width ($q_{w}$) of DSC and HD95 were calculated for each OAR in Table 1. The number of total autosegmentation cases ($N_{auto}$) and the number of outliers ($N_{outlier}$) were also provided for each software.

For the liver segmentations, Siemens not only produced the highest median DSC and lowest median HD95 but also the lowest interquartile widths (for both DSC and HD95), suggesting a good overall segmentation performance as compared to institutional contours. The Siemens outlier had a minimum DSC value of 0.894 and a maximum HD95 value of 24.34 mm, suggesting smaller discrepancies between the worst outliers and clinical contours compared to the other software tools. By contrast, Radformation produced the lowest median DSC, highest median HD95, and highest interquartile values with the minimum DSC of 0.673 and maximum HD95 of 117.79 mm.

At the time of our data collection, MIM and Siemens did not provide the stomach segmentation capability. Therefore, only stomach contours from Limbus and Radformation were evaluated. Between the two algorithms, higher median DSC, lower median HD95, and lower interquartile values were achieved by the Limbus software. If only the outliers were compared, Limbus had the higher DSC value and lower HD95 value in its worst outlier compared to Radformation.

For the kidney delineations, Limbus, Radformation, and Siemens all achieved a median DSC greater than 0.935 and median HD95 lower than 4.01 mm with DSC interquartile value below 0.031 and HD95 interquartile value below 4.01 mm, whereas the numbers were 0.891, 7.25, 0.118, and 8.07 mm for MIM, indicating significant issues with MIM's delineations for this OAR.

### 3.2 Outlier analysis

#### 3.2.1 Liver

**FIGURE 2:**
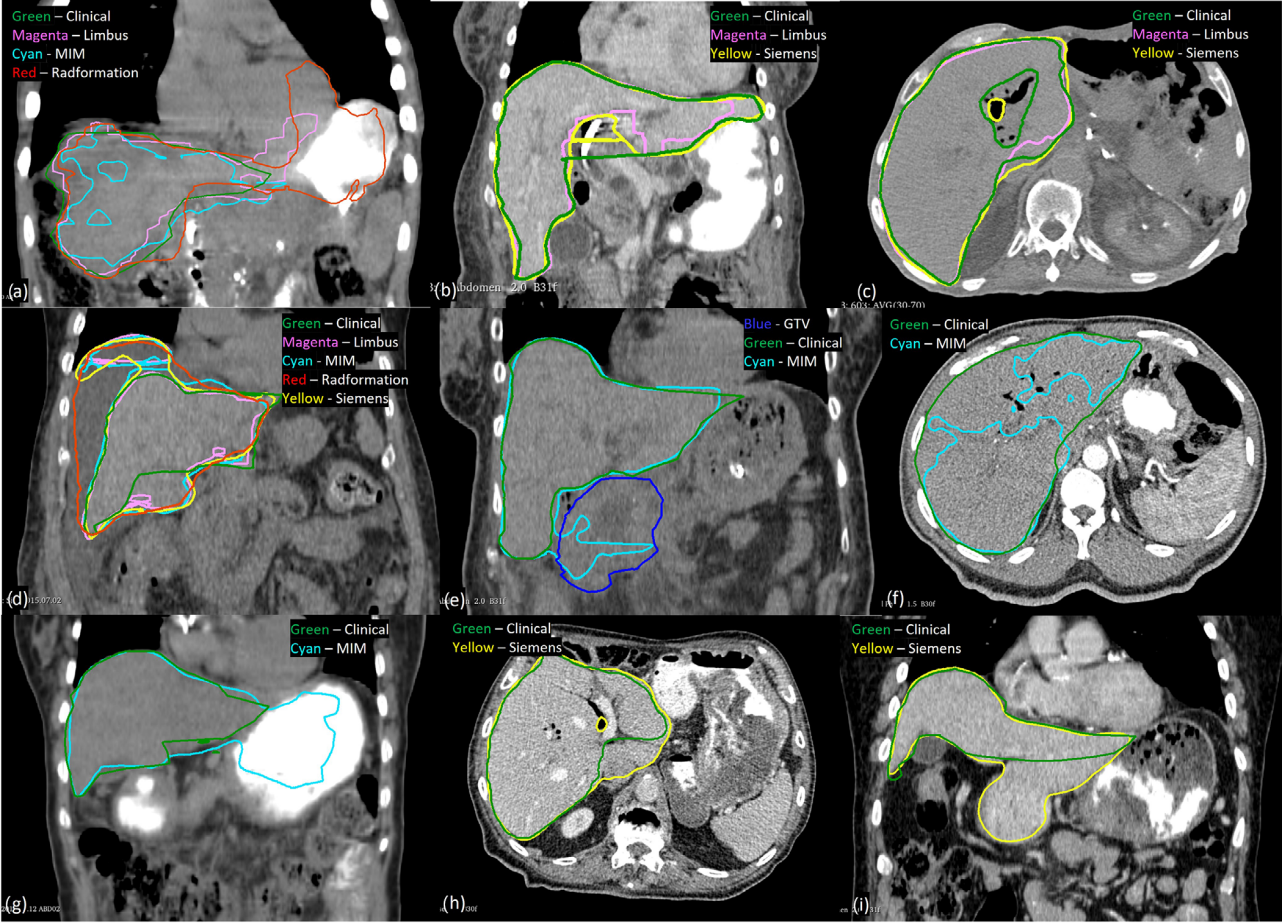
Liver autosegmentation outliers: (a) Limbus had a rugged segmentation and included part of the heart, MIM had a fragmented segmentation while Radformation contour included both stomach and heart, (b) the liver had an abnormal shape, where it extended to the left rib, and the Limbus contour was short on the left. There was an internal/external biliary drain and the resulting gas bubbles were inside the liver. They were included in the clinical contour but excluded from the Limbus and Siemens contours, (c) the Limbus contour included the pneumobilia region created by a biliary stent inferiorly while the Siemens contour excluded one individual bubble, and the clinical contour excluded the entire pneumobilia region, (d) the liver abutted ascites on the right and gallbladder inferiorly, and all autosegmentations included the entire gallbladder and some buildup fluid superiorly, (e) the MIM contour included some GTV inferiorly, (f) the MIM contour was fragmented in a region with no apparent boundaries, (g) the MIM contour included the stomach with barium contrast, (h) Siemens contour included the abutting IVC, and (i) the clinical contour missed some liver near the boundary with duodenum inferiorly while the Siemens contour included the duodenum.

The four deep learning‐based autosegmentation software exhibited different types of failures in the liver. The outlier types for liver and other abdominal OARs are summarized in Table 2. The root causes for Limbus outliers were various. 3/6 Limbus outliers were affected by the lack of soft tissue contrast or the photon starvation artifact (from barium in stomach) in the images. Figure 2a shows the case where the boundaries between the liver, heart, and stomach were blurred by the photon starvation artifact due to the barium contrast in the stomach. As a result, the Limbus contour not only had a rugged segmentation, but also extended into the heart. Biliary stent or internal/external biliary drain was observed in 4/6 Limbus outliers, and the internal device caused pneumobilia in the liver in 3/4 of those cases. There are no consensus guidelines on whether the pneumobilia region should be part of the liver delineation. It is normally included in our institutional practice, but an exception was found in 1/4 of cases with biliary stent/drain. For example, the clinical contour included the internal/external biliary drain and the gas bubble in the liver segmentation in Figure 2b, while the Limbus contour attempted to exclude the region. The opposite occurred in Figure 2c, where the clinical contour excluded the pneumobilia region superior to a biliary stent, whereas the region was included in the Limbus contour. Abnormal liver anatomy also played a role in one Limbus outlier. The liver extended to the left rib in Figure 2b, but the Limbus contour fell short on the left.

**TABLE 2:** Summary of outlier types for the abdominal OARs. HU, Hounsfield unit. The most prominent type of outliers from each software were in bold font for each OAR.

OAR	Software	Imaging	Abnormal anatomy—OAR	Abnormal anatomy—abutting tissues	Internal/external devices	Other
Liver	Limbus (*N_outlier_* = 6)	2/6 lack of soft tissue contrast. 1/6 starvation artifact from barium contrast in the stomach	1/6 abnormal liver shape	1/6 included abutting ascites and gallbladder with similar HU	**4/6 biliary stent or int/ext biliary drain (3/4 resulted in pneumobilia superiorly)**	
	MIM (*N_outlier_* = 11)			1/11 included abutting GTV 1/11 included right kidney		**5/11 fragmented MIM contours**. **5/11 contour included stomach (all 5 with barium)**
	Radformation (*N_outlier_* = 13)			2/13 included gallbladder, duodenum, or ascites with similar HU		**12/13 included abutting organ (9 with barium and 3 without barium in the stomach)**
	Siemens (*N_outlier_* = 8)	1/8 lack of soft tissue contrast	3/8 abnormal liver shape	**5/8 included gallbladder, IVC, duodenum, or right kidney**	4/8 biliary stent or int/ext biliary drain and the resulting pneumobilia	
OAR	Software	Imaging		Abnormal anatomy—OAR	Abnormal anatomy—abutting tissues	Internal/external devices
Stomach	Limbus (*N_outlier_* = 12)	1/12 lack of soft tissue contrast **10/12 imaging with barium (all 10 with incomplete stomach delineation)**		2/12 abnormal stomach anatomy 2/12 food/air in stomach	2/12 abutted GTV 2/12 Limbus included bowel	1/12 duodenal stent
	Radformation (*N_outlier_* = 25)	2/25 lack of soft tissue contrast **21/25 imaging with barium (all 21 with incomplete stomach delineation)**		5/25 abnormal stomach anatomy; 7/25 food/air in stomach	1/25 abutted GTV 7/25 Radfor‐mation included bowel	2/25 duodenal stent
OAR	Software	Delineation guideline/style	Imaging	Abnormal anatomy—OAR	Abnormal anatomy—abutting tissues	Other
Kidneys	Limbus (*N_outlier_* = 15)	**Limbus contour consistently excluded renal pelvis** 1/15 small renal cyst included in clinical contour	2/15 with rare IV contrast phase (pyelo‐graphic)	1/15 included a big renal cyst	1/15 abnormal liver location (included liver)	**10/15 contours fragmented (6 with IV contrast and 4 without)**
	MIM (*N_outlier_* = 26)		**25/26 with IV contrast (all 25 contours were fragmented)**	2/26 included a big renal cyst;	1/26 abnormal liver location (included liver)	
	Radformation (*N_outlier_* = 25)	**19/25 clinical contour inconsistency in renal pelvis**	2/25 rare IV contrast phase (pyelographic)	3/25 included partial or whole renal cyst	1/25 abnormal liver location (included liver)	
	Siemens (*N_outlier_* = 23)	**18/23 clinical contour inconsistency in renal pelvis**		4/23 included partial or whole renal cyst		

The tissues and organs abutting the liver often possess similar CT numbers with obscure boundaries, which could confuse the autosegmentations and cause delineation errors. For example, the liver abutted ascites on the right and gallbladder inferiorly in Figure 2d. All autosegmentations included the gallbladder and part of the buildup fluid superiorly. There were two MIM outliers where the MIM segmentation errors were related to the nearby tissues that possessed similar CT numbers (one tumor and one right kidney). Figure 2e shows that the MIM contour included part of the gross tumor volume (GTV) inferiorly. Other MIM outliers were more inexplicable. 5/11 Limbus outliers had fragmented contours even with clear liver boundaries, such as the one in Figure 2f. 5/11 over‐contoured and included part of the stomach, all with barium contrast present in the stomach, as seen in Figure 2g. It is worth noting that the existence of barium was not the only cause of the over‐segmentation because there were other cases where MIM correctly delineated the liver with the presence of barium.

Radformation's liver segmentation tended to include parts of the heart, stomach, or both, which accounted for 12 of the 13 outliers. This behavior can be seen in Figure 2a. Similar to MIM, the barium contrast was not the sole reason for the overextension to nearby organs, as the oversegmentation occurred in nine of the cases when barium was present and in three cases when it was absent.

The Siemens contour included gallbladder, inferior vena cava (IVC), duodenum, or the right kidney in 5/8 of its outliers. For example, the Siemens contour included the IVC in Figure 2h and the duodenum in Figure 2i. In addition, 4/8 of the Siemens and clinical contours differed in the pneumobilia region produced by a biliary stent or internal/external biliary drain. The Siemens contours tended to exclude only a small number of individual air bubbles instead of excluding the entire pneumobilia region, as demonstrated in Figure 2c,h.

#### 3.2.2 Stomach

**FIGURE 3:**
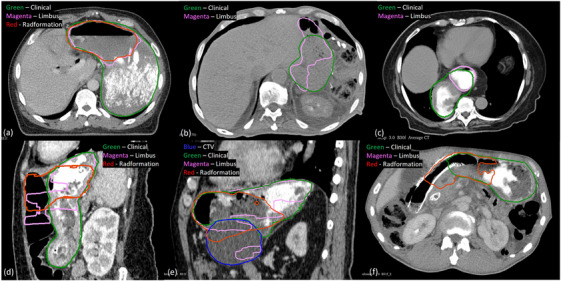
Stomach autosegmentation outliers: (a) Limbus and Radformation failed to segment the part of the stomach with barium contrast, (b) there was a lack of visible boundaries between the stomach and its surrounding tissues in the absence of barium contrast, and Limbus had fragmented segmentation, missed part of the stomach, and included part of large bowel, (c) the stomach had an abnormal location: it was located at the same level as the heart and entirely on the right side of the body. The Limbus contour missed the right and posterior part of the stomach while the Radformation contour had a very tiny volume inferior to this axial slice, (d) the stomach was filled with a mixture of food and barium, and it abutted an empty large bowel anteriorly. Both Limbus and Radformation included the large bowel superiorly and missed the entire inferior stomach where it flexed to the right. Limbus also produced fragmented segmentation, (e) the stomach abutted the CTV inferiorly. Both Limbus and Radformation under‐segmented the stomach and included part of the CTV, and (f) the Radformation contour extended to the duodenum stent and had incomplete delineation on the left while Limbus did not segment the stomach at the level of the stent.

The stomach is a challenging OAR for the autosegmentation solutions to delineate. Firstly, the CT imaging was often done with barium in order to create contrast against nearby tissues, but both Limbus and Radformation struggled when barium was present in the images. As seen in Figure 3a, both Radformation and Limbus contours missed most of the stomach that contained the barium contrast. The barium was the most prominent factor in segmentation errors in Radformation and Limbus contours. Out of the 25 Radformation outliers, barium was used in imaging in 21 of them, and incomplete stomach segmentation was observed in all 21 cases. Similarly, barium was present in 10/12 Limbus outliers and all 10 missed non‐trivial volume of the stomach. On the other hand, for imaging done without the barium, the numerous abdominal organs share similar CT numbers with indistinct boundaries, which was also challenging for the autosegmentation software. For example, there were no visible boundaries in many axial slices between the stomach and liver in Figure 3b. As a result, a fragmented segmentation was produced by Limbus which missed some of the stomach and included some of the large bowel anteriorly.

In addition, the shape, size, and location of the stomach can vary. Abnormal liver anatomy was found in 2/12 Limbus outliers and 5/25 Radformation outliers. For example, the stomach in Figure 3c was located at the same level as the heart and entirely on the right side of the body, posterior to the heart and the liver. The Limbus contour did not segment the right and posterior part of the stomach while the Radformation contour was not seen in the image at all because it only segmented a very small volume inferior to the shown slice.

Besides the barium, it was not uncommon to see air and/or food in the stomach, and the mixture of barium, food, and gas in the stomach could create great Hounsfield unit (HU) heterogeneity in the stomach, making it difficult for the autosegmentation tools to accurately delineate. The content in the stomach was believed to be a factor in the segmentation failures in 2/12 Limbus outliers and 7/25 Radformation outliers. In Figure 3d, the stomach not only was filled with a mixture of barium and food, but also abutted the empty large bowel anteriorly. Both Limbus and Radformation contours stopped short inferiorly and included the large bowel anteriorly with the limbus contour fragmented again. Because gas is often present in the bowel, just like in the stomach, it was considered another factor in segmentation failures. As shown in Table 2, 2/12 Limbus outliers and 7/25 Radformation outliers included part of the small or large bowel.

Apart from challenges from the imaging (with and without barium), abnormal shape of the stomach, food and air in the stomach, and the abutting bowel, the duodenum stent, and a tumor in the region could also cause segmentation discrepancies. Figure 3e shows that both Radformation and Limbus contours included part of the clinical target volume (CTV) inferior to the stomach. The Radformation contour extended a little further to the right along the duodenum stent than the clinical contour while the Limbus contour missed the inferior stomach at the level of the stent entirely in Figure 3f.

#### 3.2.3 Kidneys

**FIGURE 4:**
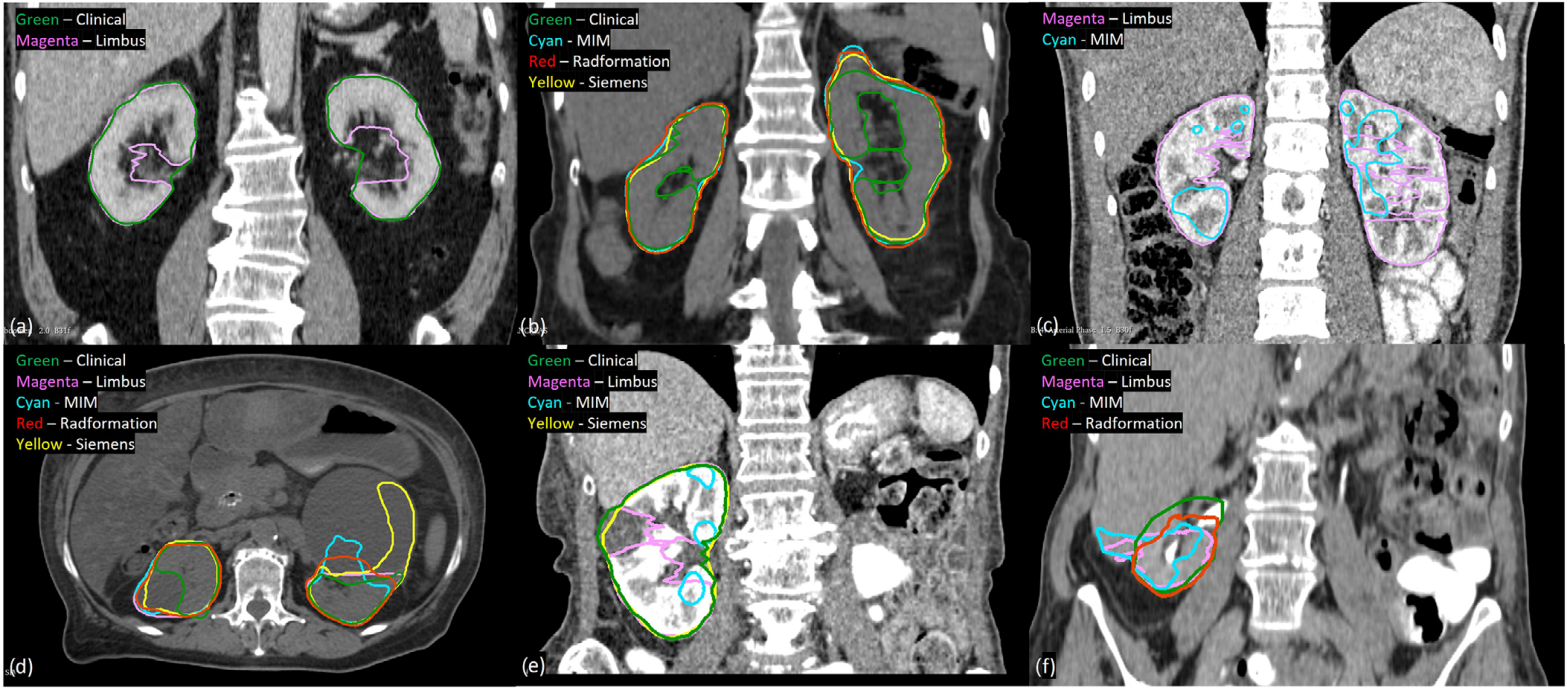
Kidney autosegmentation outliers: (a) Limbus's delineation guideline does not include the renal pelvis while the clinical contour did for the patient, (b) Radformation and Siemens delineation guidelines include the renal pelvis while the clinical contour did not for the patient. The adrenal gland superior to the left kidney was also included by the autocontours, (c) fragmented Limbus and MIM segmentations in the presence of IV contrast, (d) the cysts abutting the left and right kidneys were not a part of the clinical contours while all autocontours included partial or the whole cyst. The images were not acquired with IV contrast, (e) the small cyst was included in the clinical contour, whereas it was excluded from the Limbus contour, and (f) the segmentation of the right kidney was negatively affected for all vendors by the enlarged liver that extended far inferiorly and abutted the right kidney with very similar HU values. The images were acquired in the pyelographic phase of IV contrast, which could not provide enhancement of renal parenchyma.

The CT images for the kidneys were often acquired with an intravenous (IV) iodinated contrast media, providing good contrast and clear boundaries against nearby organs and potential cysts. The most prominent reason for kidney autosegmentation outliers was the difference in contouring guidelines. The RTOG consensus guidelines for the kidneys recommend the segmentation of the entire kidney, including the renal pelvis.^21^ MIM, Radformation, and Siemens followed the RTOG guidelines while Limbus consistently excluded the renal pelvis. As for our clinical contours, the consensus guidelines were not strictly followed in some cases. As a result, 19/25 of the Radformation and 18/23 of the Siemens cases were found with discrepancies in the renal pelvis, as shown in Figure 4a,b. Another major outlier type is the fragmented contours for MIM when IV contrast is present. Every single one of the 25 MIM outliers in the presence of IV contrast had fragmented contours, exemplified by Figure 4c. Broken contours are also a major problem for Limbus. However, unlike MIM, the IV contrast was not the only cause of fragmented contours in Limbus as IV contrast was present in only 6/10 of its fragmented contours.

Renal cysts could lead to inaccurate delineations as well. The contouring guidelines do not specify whether renal cysts should be included in the kidney segmentation.^21^ Institutionally, the small cysts are typically included in the delineation while the big cysts with clear boundaries are not. For example, both kidneys abutted a renal cyst with similar HUs in Figure 4d. The cysts were not part of the clinical contours, but all the commercial solutions included partial or the whole cyst in the delineations. On the other hand, the small cyst in Figure 4e was included in the clinical contour. The Siemens contour also included the cyst despite it possessed a different HU level than the renal parenchyma, whereas the Limbus contour did not include the cyst. Imaging can also affect the quality of the segmentations. The most common CT protocol for renal masses is a non‐enhanced phase followed by a nephrographic phase, which occurs about 90–120 s after the contrast injection and could show the complete enhancement of the renal parenchyma.^22^ However, we have found cases where the images were either acquired at a delayed time and ended up in the pyelographic phase, causing 2/15 Limbus outliers and 2/25 Radformation outliers, or acquired without any contrast. Without the enhancement of the renal parenchyma, it made it more difficult for the software to differentiate the kidneys from the abutting tissues like renal cysts in Figure 4d and the liver in Figure 4f.

## 4 DISCUSSION

Commercial deep learning‐based autosegmentations have been reported to have many advantages over manual contours, including higher geometric accuracies and time‐saving.^11^ However, there is a lack of failure analysis for these tools, which is a critical step in the iterative process of implementing such tools in the clinic. This work described the segmentation outliers in the abdominal OARs and summarized them into six categories. It would help physicians be prepared for potential segmentation failures when they encounter images or patients with known risks of segmentation failures. It is even possible to avoid spending more time on contouring by not using the autosegmentation software in those situations. The vendors could also use the results provided in this work to include more outliers in the training data and build a better algorithm for each OAR.

The outlier analysis conducted in this work has uncovered some root causes for the segmentation discrepancies, and the most prominent ones are summarized for each software in Table 3. For the Limbus software, the fact that an internal device (biliary stent or biliary drain) was the top cause of outliers suggested that the Limbus's liver segmentations were free from other major modes of failures, and the users can expect a decent auto‐contour that requires little editing. However, the Limbus segmentations of the stomach missed a significant portion of the organ when barium contrast was present, which made us to believe that there was a serious lack of representation of barium contrast imaging during the training of the algorithms. With regard to the segmentation of the kidneys, Limbus appeared to have followed a set of contour guidelines different from the RTOG consensus guidelines and routinely excluded the renal pelvis in the kidney contours, and the users need to be aware of this difference when using the software.

**TABLE 3:** Summary of the most prominent auto‐segmentation discrepancies for each software.

OAR	Limbus	MIM	Radformation	Siemens
Liver	Biliary stent/drain & resulting pneumobilia	Fragmented Contours Included stomach (all occurred with barium contrast but not the only factor)	Significant over‐extension into heart and stomach even with barium contrast	Biliary stent/drain & resulting pneumobilia Inclusion of kidney, IVC, duodenum, and gallbladder
Stomach	Significant portion of stomach unsegmented due to barium contrast		Significant portion of stomach unsegmented due to barium contrast	
Kidneys	Guideline difference (renal pelvis excluded) Broken contours	Severely fragmented contours caused by IV contrast	Benefit: Followed RTOG contouring guidelines more consistently than manual contour	Benefit: Followed RTOG contouring guidelines more consistently than manual contour

MIM produced fragmented contours in the liver and the kidneys. This type of failure is clinically unacceptable because the users cannot make minor edits to reduce contouring time. These failures could not be blamed solely on the lack of contrast images in the training dataset, the solution to fragmented contours of any software, regardless of the root causes, must involve the improvement in the cost function of the deep learning model or the model itself that would increase the penalization of large‐scale fragmentation and discontinuity of contours in consecutive axial slices.

Radformation caused significant overextension into the heart and stomach in its liver segmentation, and one could not predict when they would occur and how much over‐segmentation there would be because the Radformation contour could not only disregard the vast difference in CT numbers and include both the stomach with barium, but also randomly contour through homogeneous regions, as demonstrated in Figure 2a. The liver and stomach contours also revealed another flaw in the software. The software could produce overlapping liver and stomach contours, but neither contour was rejected by the software, indicating insufficient secondary checks in the software. When it comes to the kidneys, Radformation showed one of the benefits of auto‐segmentation. It could produce contours that follow the RTOG contouring guidelines more consistently than the manual contours. The inclusion of the pelvis is always expected, and the small vessels and fissures inside the kidney will not be excluded.

Siemens did not show major problems with contrast imaging and did not produce broken contours in its contours in the abdominal region. Similar to Radformation, it also exhibited an advantage in consistently following the contouring guidelines in its kidneys. When using the software for liver segmentation, the users ought to check the boundaries with IVC, duodenum, gallbladder, and kidneys.

In this work, we tried to investigate the most prominent failure modes of each software for each OAR, but it is important to note that some root causes cannot be uncovered until the existing problems are resolved. For example, the Radformation's stomach contour may include some bowel if the bowel is located at a similar level as the stomach, but the extent of this risk won't be accurately assessed until the problem with barium contrast imaging is resolved. Similarly, MIM's liver segmentation also tended to include the stomach in the presence of barium, but the barium itself could not explain the oversegmentation, and the other factor(s) may not be revealed until MIM works for contrast images. In addition, it is always challenging to determine the boundary between the stomach and duodenum, but the assessment of the inferior end of the stomach delineation could not be performed until the issue with barium contrast was resolved for both Limbus and Radformation software.

The outliers analyzed in this work were from the geometric evaluations based on two metrics, DSC and HD95, while dosimetric information, contour editing time (or total contouring time), and physician's ratings were not collected in the work. Specifically, the outliers were selected using the distribution of the DSC and HD95 scores of each individual software instead of setting a single DSC and HD95 threshold for all software tools for a given OAR. As a result, the number of outliers extracted from a software tool was not related to how well it performed compared to other software solutions. The reason why the outliers were selected in this fashion was that the clinical acceptability of a contour cannot be determined by thresholding of geometric evaluation scores. Although it may seem unfair for the software tools that achieved better geometric evaluation scores (higher DSC and lower HD95) and may leave some problems undiscovered for the tools that scored relatively worse in the evaluations, it allowed us to identify the biggest segmentation errors for each software. It is also why the selection of multiplier length w in the determination of outlier thresholds only depended on how many outliers were needed to reveal the biggest failure modes. In this work, the failure modes were limited to the six categories we analyzed. Patient information such as age and gender were not considered. In addition, although the failure mode directly related to the use of contrast agents was identified, other CT image characteristics, such as the imaging device and CT protocol, were not investigated. Last but not least, it is important to note that the autocontours evaluated in this study may not reflect the quality of the software now, as the autocontours were collected between June 2021 and July 2022. Since then, all software tools have been upgraded, and Limbus AI has been acquired by Radformation, Inc.

## 5 CONCLUSION

We performed outlier analysis for the autosegmentation offered by Limbus Contour, MIM ProtegeAI, Radformation AutoContour, and Siemens Syngo.via for the abdominal OARs (liver, stomach, and kidneys). The commercial software solutions showed a known benefit of autocontouring, where they could follow the consensus contouring guidelines more consistently than the manual contour. However, there were also some major segmentation failures, such as fragmented contours, erroneous contours in the presence of contrast images, and significant overextension into abutting organs. We believe that the causes of outliers summarized in this work can help the vendors improve their autosegmentation software and inform the users of potential delineation failures when using the tools.

## AUTHOR CONTRIBUTIONS

The authors would like to thank Dr. Mingdong Fan for his data analysis and the writing of the manuscript, Dr. Tonghe Wang and Dr. Yang Lei for their contributions to data processing and initial data analysis, Sean Dresser, Beth Bradshaw Ghavidel, Dr. Richard L.J. Qiu, Dr. Jun Zhou, Oluwatosin Kayode, Dr. Jeffrey D. Bradley for their assistance in data collection and manuscript editing, Dr. Pretesh R. Patel, and Kirk Luca for their insight on contouring and assistance in manuscript editing, and Dr. Xiaofeng Yang and D. Justin Roper for their supervision of the project and manuscript editing.

## CONFLICT OF INTEREST STATEMENT

The authors declare no conflicts of interest.

## ETHICS STATEMENT

This study was approved by the Institutional Review Board of Emory University (STUDY00006087).
